# The zebrafish ventricle: A hub of cardiac endothelial cells for *in vitro* cell behavior studies

**DOI:** 10.1038/s41598-017-02461-1

**Published:** 2017-06-02

**Authors:** Chinmoy Patra, Zacharias Kontarakis, Harmandeep Kaur, Amey Rayrikar, Debanjan Mukherjee, Didier Y. R. Stainier

**Affiliations:** 1 Agharkar Research Institute, Developmental Biology, Pune, India; 2 Max Planck Institute for Heart and Lung Research, Department of Developmental Genetics, Bad Nauheim, Germany; 3 Max Planck Institute for Heart and Lung Research, Department of Pharmacology, Bad Nauheim, Germany

## Abstract

Despite our increasing understanding of zebrafish heart development and regeneration, there is limited information about the distribution of endothelial cells (ECs) in the adult zebrafish heart. Here, we investigate and compare the distribution of cardiac ECs (cECs) in adult mouse and zebrafish ventricles. Surprisingly, we find that (i) active coronary vessel growth is present in adult zebrafish, (ii) ~37 and ~39% of cells in the zebrafish heart are ECs and cardiomyocytes, respectively, a composition similar to that seen in mouse. However, we find that in zebrafish, ~36% of the ventricular tissue is covered with ECs, i.e., a substantially larger proportion than in mouse. Capitalising on the high abundance of cECs in zebrafish, we established a protocol to isolate them with high purity using fluorescent transgenic lines. Our approach eliminates side-effects due to antibody utilisation. Moreover, the isolated cECs maintained a high proliferation index even after three passages and were amenable to pharmacological treatments to study cEC migration *in vitro*. Such primary cultures will be a useful tool for supplementary *in vitro* studies on the accumulating zebrafish mutant lines as well as the screening of small molecule libraries on cardiac specific endothelial cells.

## Introduction

The morphological diversity and cell surface protein heterogeneity of endothelial cells (ECs) in different organs of the body is known since the early 1990s^1, 2^. Despite increasing evidence for the importance of organ specific ECs in organ development^3, 4^, little is known about the involvement of cardiac endothelial cells (cECs) in heart development, growth, and homeostasis^5^ and subsequently their contribution to cardiac pathophysiology. Earlier reports have suggested that mouse hearts comprise ~50% cardiomyocytes (CMs), ~27% cardiac fibroblasts and a minor fraction of ECs^6, 7^, while more recent data estimate ~31% CMs and ~43% ECs^8^. Although zebrafish is a very powerful model organism for heart development and regeneration studies, until today the cellular composition of the zebrafish heart has not been examined.

The diversity of ECs in different organs certainly represents their specific functions and requirements in different tissues; for example, ECs residing among stromal cells in the bone marrow actively participate in long-term multilineage hematopoiesis^1^. In addition, bone marrow capillaries are fenestrated, which might facilitate the trafficking of hematopoietic and mature blood cells^1^. In contrast, in the brain microvasculature, well-developed tight junctions between ECs ensure the selective transport between the blood and central nervous system^1^. This EC specialization takes place in the microenvironments of the different organs during their development^9^. Thus, the study of a single EC type (e. g. human umbilical ECs) fails to sample the tissue specific peculiarities of ECs, an important goal for treating pathologies associated with particular organs. A few attempts towards this direction have utilised immunomagnetic cell enrichment to isolate endothelial cells from mammalian organs for *in vitro* studies^2, 10^, but not from zebrafish, an important *in vivo* model for studying organ development and regeneration. Here, we report the high abundance of cECs in the adult zebrafish ventricle and exploit this feature to establish cEC isolation and culturing method. Using tissue specific reporter lines, flow cytometry, EdU incorporation assay and immunohistochemical analysis we show that (i) coronary vessels continuously grow in adult zebrafish, (ii) the relative surface area of the ventricle covered by ECs is larger in zebrafish than in mouse, (iii) ~37 and ~39% of cells in the zebrafish heart are ECs and CMs, respectively, (iv) highly pure primary cEC cultures can be obtained from isolated hearts, and (v) cECs are highly proliferative and responsive to small molecules *in vitro*.

## Methods

### Zebrafish maintenance

Wild-type and transgenic (*Tg(fli1a:EGFP)* ^*y1*^, *Tg(kdrl:Hsa.HRAS-mCherry)* ^*s896*^, *Tg(kdrl:EGFP)*, *Tg(myl7:GFP)* ^*twu34*^) zebrafish (*Danio rerio*) were maintained at 28 °C as previously described^11–15^. 25–35 embryos were grown in a 3.5 liter tank from 6 dpf to 25 dpf. Immediately after, only the largest animals were selected and 12–15 animals were maintained in a 3.5 liter tank for further experiments. All zebrafish husbandry was performed under standard conditions in accordance with institutional (MPG) and national ethical and animal welfare guidelines.

### Confocal microscopy of whole mount hearts

For confocal microscopy, hearts were isolated from tricaine-anesthetized and sacrificed adult animals. Freshly isolated PBS rinsed hearts were immediately mounted in 1% low-melting point agarose/PBS (0.01% tricaine) in a glass bottom dish. Confocal sections covering the entire heart were imaged from the ventral and dorsal sides on a Zeiss LSM780 or Leica SP8 and processed to obtain maximal intensity projections. Vascular density was analysed using ImageJ/Fiji software.

### Immunohistochemistry and histological analysis

For the coronary vessel distribution studies, freshly isolated cardiac ventricles from 8 months old wild-type mice and *Tg(kdrl:Hsa.HRAS-mCherry)* ^*s896*^ zebrafish were embedded in OCT medium (Sakura Finetek, USA). 10 µm thick sagittal cryosections were prepared in a Leica CM3050S cryostat. We used anti-CD31 and anti-sarcomeric-α-actinin to visualise ECs and CMs respectively in sagittal cryosections of mouse hearts. Similarly, sagittal sections through the hearts of *Tg(kdrl:Hsa.HRAS-mCherry)* ^*s896*^ fish which show mCherry expression in the plasma membrane of vascular ECs were immunostained for mCherry and CM specific α-actinin/with Alexa-488 conjugated phalloidin to stain cardiac tissue. Immunohistochemistry was performed as previously described^16^. Immediately after the blocking step, samples were incubated overnight with primary antibodies [mouse anti-sarcomeric α-actinin, 1:400 (Sigma); rat anti-CD31, 1:100 (BD Biosciences); and rabbit anti-mCherry, 1:500 (Clontech); rabbit anti-EGFP, 1:500 (Novus biologicals)] at 4 °C. To detect primary immune complexes, Alexa 488- or Alexa 594-conjugated antibodies (1:400; Molecular Probes) were used. EdU detection was performed after completion of immunostaining of the cells, following manufacturer’s instructions (Molecular Probes™). For phalloidin staining, cells were incubated with rhodamine/Alexa-488 conjugated phalloidin (1:50; Molecular Probes) together with the primary antibody. 4′,6′-diamidino-2-phenylindole (DAPI; Sigma) (0.5 µg/ml water) was used to stain nuclei. Confocal optical sections were captured using a Leica SP8 or a Zeiss LSM 700 laser scanning microscope. ImageJ/Fiji software was used to analyze cardiac tissue area covered by ECs.

### Coating for cell culture

After the 4 h pre-plating step, the cell suspension was cultured in coated plastic bottom culture dish or on coated glass coverslips (B 12 mm, Karl Hecht GmbH, Germany), placed in 24-well tissue culture plates. 250 µl and 150 µl of fibronectin solution (10 µg/ml PBS) (PromoCell, Germany) was used to coat each well of a 24 and 48 well plate, respectively, with a 2 h incubation at 37 °C. For glass coverslips, they were placed in the well of a 24 well plate and treated twice with 70% ethanol for 5 minutes each and once with 100% ethanol for 3 minutes. Air dried glass coverslips were then incubated with 100 µl fibronectin solution (10 µg/ml) for 2 h at 37 °C. The coating solution was afterwards aspirated and coverslips were air dried for 30 minutes.

### Cardiac endothelial cell isolation and culture

Animal experiments were approved by the local Committee for Care and Use of Laboratory Animals and follow the Guide for the Care and Use of Laboratory Animals published by the US National Institutes of Health. Isolated hearts (10–15 for each isolation) from 7–8 month post fertilisation (mpf) old animals were washed in an ice cold heparin solution (10U/ml in PBS) to remove blood (see Supplementary Fig. S3). Distinct parts of the cardiac tissue (ventricle/atrium/bulbus arteriosus) were collected in 1.5 ml centrifuge tubes containing ice cold PBS supplemented with 5 mM glucose. Harvested heart tissue was centrifuged at 300 g for 3 minutes at 4 °C to pellet. The tissue was then transferred to a round bottom 10 ml corex glass tube along with a magnetic stir bar and 1.5 ml digestion cocktail in DMEM [collagenase type II (250 U/ml), collagenase type IV (300 U/ml) and DNase I (30 µg/ml)]. Subsequently, the corex glass tube was transferred in a 32 °C water bath (with stirring 200 to 300 rpm) and incubated for 1 minute. After that, the corex glass tube was removed from the water bath and left at room temperature for the tissue to settle on the bottom (1 minute tissue sedimentation). The supernatant was discarded to remove remaining blood cells from the sample. This wash step was followed by a series of digestion steps (4–6 times) each with 1.5 ml digestion buffer. All digestion steps involved 15 minutes of digestion followed by 3 minutes sedimentation. After each sedimentation step, the supernatant containing the cells was collected in a 15 ml falcon tube containing 2 ml ice-cold FBS kept on ice. The tubes containing the cell suspension in FBS were centrifuged at 300 *g* for 5 minutes at 4 °C. The supernatant was discarded and the cell pellets were washed by re-suspending in EGM2 medium and again centrifuged at 300 *g* for 5 minutes at 4°C. Supernatant was discarded and cell pellet was resuspended in prewarmed EGM2 medium with all supplements and 10% FBS (30 °C, 1 ml per 5 hearts). The cell suspension was pre-plated on 3.5 cm cell culture dishes (2.5 ml cell suspension/dish) and incubated for 4 h at 28 °C with 5% CO_2_. During this pre-plating step, non-CMs and non-endothelial cells attached to the cell culture dish, enriching the CM and endothelial cell population in suspension. The supernatant from the pre-plating step was transferred to a fibronectin pre-coated culture plate and cultured for 18 h at 28 °C with 5% CO_2_. After 18 h, the CM enriched supernatant was discarded and cells were washed with EGM2 medium. Attached ECs were maintained in EGM2 medium with all supplements and 10% FBS (culture medium). Cells were grown to 90% confluence before passaging. To passage, cells were detached by trypsinization (0.05% trypsin) for 5 minutes at 28 °C and then diluted with culture medium. The cell containing medium was centrifuged at 300 g for 5 minutes and the pellet was washed once with culture medium, before re-suspending and re-plating the cells on new fibronectin coated culture dishes.

### FACS analysis

Cells from 7–8 mpf *Tg(kdrl:EGFP)* ^*s843*^ and *Tg(myl7:GFP)* ^*twu34*^ ventricles, respectively marking the ECs and CMs with EGFP were isolated as describe above. For each experiment, cells from a pool of 10–12 ventricles were resuspended in ice cold PBS and analyzed for live and dead cell population and cECs and CMs cell population in the live cell fraction. For live and dead cell analysis, cell suspensions were incubated with DRAQ and 7AAD for 10 minutes at RT, and subsequently the stained cells were washed twice with PBS and analyzed using a BD FACS Canto II.

### Cell cycle activity

In each well of a 24 well plate, 1 × 10^5^ cECs either immediately after isolation or from a 2^nd^ passage were seeded on fibronectin coated glass coverslips. After 40 h, cells were serum starved for 12 h or cultured in cell culture medium containing 0.5 or 10% FBS. Subsequently, EdU (500 nM) was added to the culture medium. 12 h later, cells were fixed with 4% PFA and processed for staining. Cell cycle activity was quantified by counting at least 500 cECs from at least 5 randomly chosen microscopic fields per experiment. For the *in vivo* cell cycle activity assay, 6 mpf *Tg(fli1a:EGFP)* zebrafish were injected intraperitoneally with 10 µl of 10 mM EdU (Molecular Probes™, C10340) (every 24 h, 3 times in total), and hearts were collected and fixed in 4% PFA 24 h after the last injection. Whole mount EdU detection was performed after completion of immunostaining with anti-GFP antibody following manufacturer’s instructions (Molecular Probes™). DAPI; Sigma (0.5 mg/mL water) was used to stain nuclei. For quantification, the number of *fli1a*: EGFP+ EdU+ cells was calculated per heart.

### Gene expression analysis

Total RNA was isolated from cardiac ventricles (pool of 3) and endothelial cell-enriched culture after a 2^nd^ or 3^rd^ passage using the RNeasy mini kit (Qiagen, Germany). 500 ng of total RNA was subjected to cDNA synthesis using an oligo dt primer (Invitrogen) following standard protocol. Quantitative RT-PCR analysis was performed in a CFX96 Touch™ Real-Time PCR Detection System (Bio-rad) using DyNAmo ColorFlash SYBR Green qPCR mix (Thermo Scientific™) and the primer pairs mentioned in Supplementary Table S1. Gene mRNA expression levels relative to α-tubulin were calculated using the ΔCt method (Supplemental Table S2). All reactions were run in triplicate.

### Wound healing assay

In each well of a 48 well plate, 0.5 × 10^5^ cardiac ventricular ECs from a 2^nd^ or 3^rd^ passage were seeded with 10% FBS and left to grow into a confluent monolayer. To assay the basal cell migration ability of cardiac ventricular ECs, confluent monolayer cells were serum starved for 6 h and subsequently a wound on the monolayer was created using a p20 pipette tip and the cell free area was measured 4 and 16 h after the scratch. To study cEC response to small molecules and growth factors treatment, cells were cultured with 10% FBS for 48 h and then serum and VEGF starved for 6 h. Subsequently, cells were treated with DMSO (control), a medium containing VEGF (same concentration as in EGM2), or with paclitaxel (12 nM final concentration). The cell monolayer was scratched 12 h post treatment and the cell free area was measured 4 and 16 h after the scratch. Initial wound area was set using images 4 h post scratch. Subsequently, the same region was analyzed and compared with the initial wound. ImageJ/Fiji software was used to analyze the cell free area reduction over 12 h.

### Endothelial cell morphology

Cells from the three chambers (atrium, ventricle, bulbus arteriosus (BA)), and another highly vascularized organ, the gills, were isolated, cultured for 3 days and subsequently fixed and immunostained with anti-EGFP (ECs), and DAPI (nuclei). Cell circularity was measured with the ImageJ/Fiji software and we considered cells as circular or elongated when value was >0.5 and ≤0.5, respectively.

### Data analysis

All data are expressed as the mean ± SEM of ≥ three independent experiments. One way ANOVA followed by Bonferroni’s post-hoc test (GraphPad Prism) was performed to evaluate statistical significance of differences. P < 0.05 was considered statistically significant.

## Results

### Active coronary vessel growth in adult zebrafish

**Figure 1:**
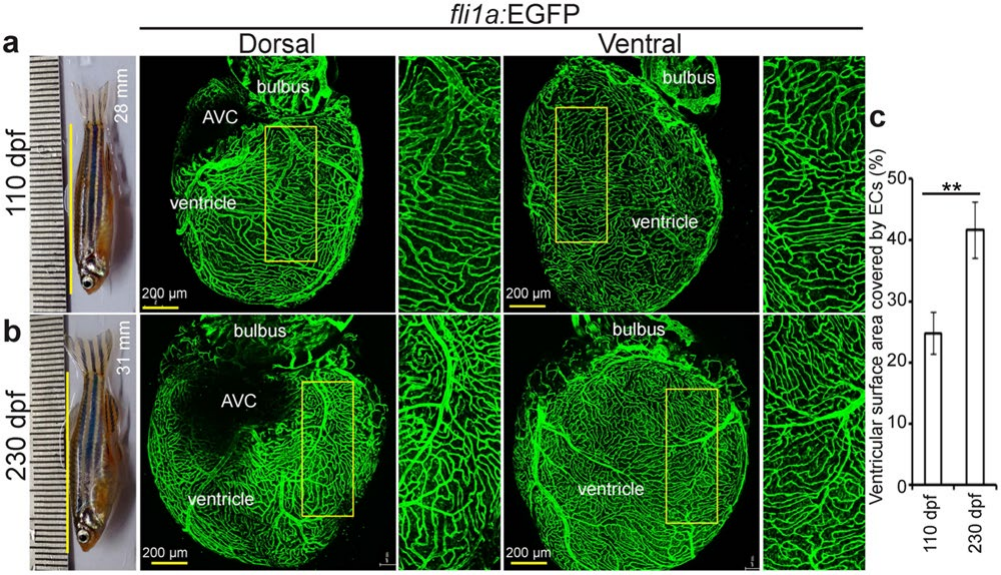
Zebrafish coronary vessels continue to grow after 3 months post fertilization. (**a,b**) Representative brightfield images of adult zebrafish and maximum intensity projections of confocal images of freshly isolated hearts from 110 (**a**) and 230 (**b**) dpf *Tg(fli1a:EGFP)* animals. Green cells have pan-endothelial identity. (**c**) Quantification of cardiac ventricular surface area covered by endothelial cells (n = 3 of each stage, mean ± SEM). One way ANOVA followed by Bonferroni’s post-hoc test (GraphPad Prism) was performed to evaluate statistical significance of differences. P < 0.05 was considered statistically significant. ** corresponds to P < 0.05. AVC- atrioventricular canal, dpf- days post fertilization.

Recently, Harrison *et al*. suggested that coronary vessel density in adult zebrafish continues to increase^17^. Here, we compared vessel density between 110 and 230 days post fertilisation (dpf) in *Tg(fli1a:EGFP)* ^*y1*^ zebrafish, using confocal imaging of whole mount freshly isolated cardiac ventricles and also explored cell cycle activity in coronary ECs at 6 months post fertilisation (mpf). We found around 70% increments in vessel density (from 24.71 ± 3.39% to 41.56 ± 4.58%) during that period (Fig. 1). Cell cycle activity in cECs was assessed by *in vivo* EdU incorporation. In each heart around 100 coronary ECs incorporated EdU after 3 consecutive EdU injections every 24 h (see Supplementary Fig. S1a–e). Thus, our data indicate that coronary endothelial cells are proliferative and those coronary vessels continue to grow in adult zebrafish.

### Endothelial abundance in the zebrafish cardiac ventricle

**Figure 2:**
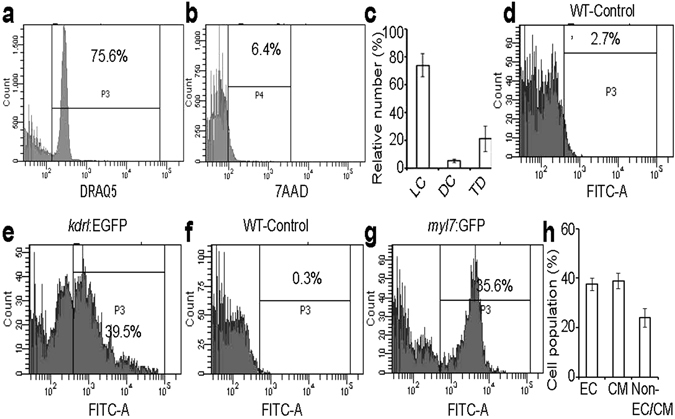
Flow cytometry analysis of cells isolated from adult zebrafish ventricles. (**a–g**) Live cell fraction (**a**), dead cell fraction (**b**), quantitative analysis of live cells (LC), dead cells (DC), and tissue debris fraction (TD) in the cell preparation samples (n = 3, mean ± SEM) (**c**), EGFP positive (endothelial cells) fraction in DRAQ5 positive live cell population of WT and *Tg(kdrl:EGFP)* fish ventricles (**d,e**), and EGFP positive (cardiomyocytes) fraction in DRAQ5 positive live cell population of WT and *Tg(myl7:EGFP)* fish ventricles (**f,g**). (**h**) Quantification of different cell types; endothelial cells (EC) and cardiomyocytes (CM) constituted 37.36 ± 2.49% and 38.80 ± 3.17% of the DRAQ5 positive cell population originating from cardiac ventricles, respectively (n = 3, mean ± SEM). WT- wild-type (non-transgenic).

**Figure 3:**
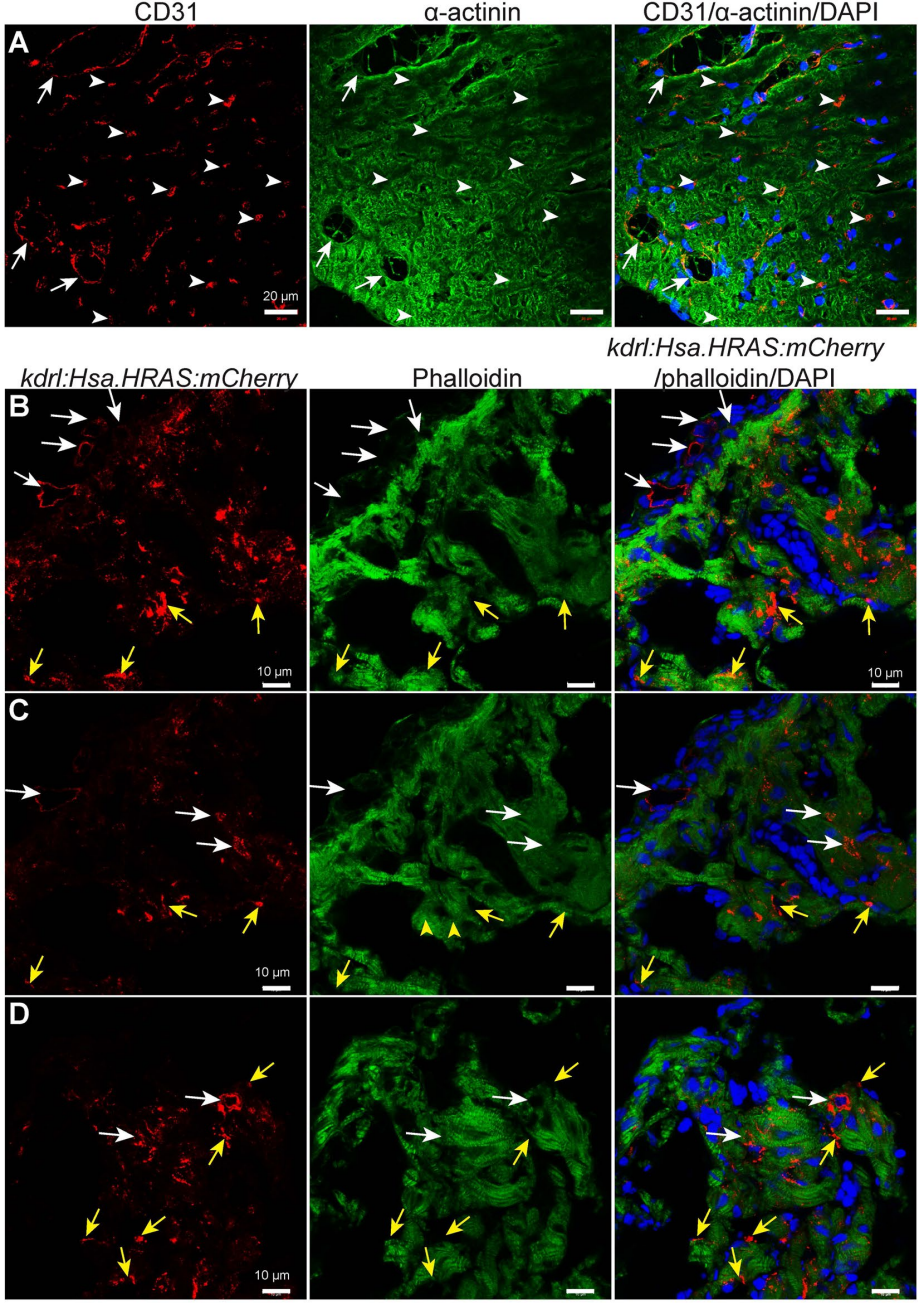
Endothelial cell distribution in 8 months old mouse and zebrafish ventricles. (**A**) Representative confocal images of sagittal cryosections through 8 months old mouse hearts stained for α-actinin (green; marking cardiomyocytes), CD31 (red; marking the cell membrane of endothelial cells) and DAPI (blue, staining the nuclei). White arrows and arrowheads point to bigger coronary vessels (visible lumen) and capillaries (lumen not visible) in cardiac tissue, respectively. (**B–D**) Representative confocal images of sagittal cryosections through 8 mpf *Tg(kdrl:Hsa.HRAS-mCherry)* zebrafish hearts stained for phalloidin (green; marking cardiac tissue), mCherry (red; marking the cell membrane of endothelial cells) and DAPI (blue; staining the nuclei). White arrows point to bigger coronary vessels (visible lumen). Yellow arrows point to endocardial cells in the cardiac tissue. ‘B’ and ‘C’ images show different optical planes of the same tissue section.

In order to quantify the different cell populations in the adult zebrafish heart, cells from the heart from 7–8 mpf *Tg(kdrl:EGFP)* ^*s843*^ and *Tg(myl7:GFP)* ^*twu34*^ zebrafish, respectively marking the ECs and CMs with EGFP, were isolated and analysed by FACS. In the live cell fraction (93.49 ± 1.14% live and 6.50 ± 1.14% dead cells in the total cell population) (Fig. 2a–c), we identified 37.36 ± 2.49% of cells as ECs and 38.80 ± 3.17% as CMs, leaving ~24% for other cell types (Fig. 2d–h), a composition similar to that recently reported in the mouse heart^8^. In the next step, we sought to compare the EC distribution in 8 months old mouse and zebrafish ventricles. We found that in zebrafish, large coronary vessels are mostly localized in the outer cortical layer while smaller vessels are distributed in the compact and trabecular layers (Fig. 3). In addition, the cEC density is many folds higher in zebrafish; 35.66 ± 3.76% versus 7.38 ± 1.32% of ventricular cardiac tissue is covered by cECs in zebrafish versus mouse (Fig. S2). Taken together, our data suggest that CMs and ECs constitute the majority of cardiac cells in zebrafish, similar to findings in mouse. However, the relative ventricular surface area covered by ECs in zebrafish is larger than that in mouse.

### Endothelial cell isolation for *in vitro* studies

**Figure 4:**
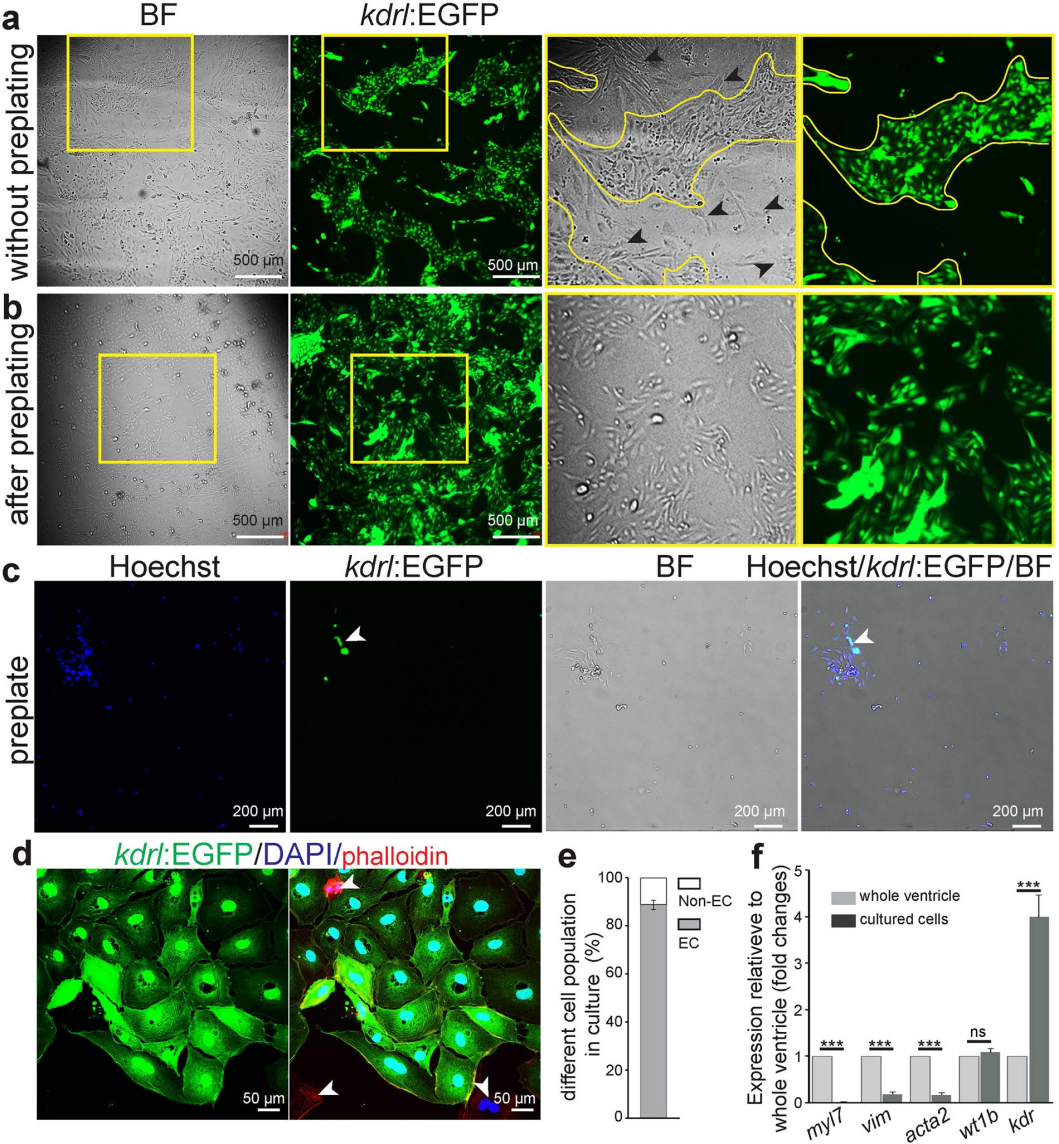
Culture of ventricular endothelial cells isolated from adult *Tg(kdrl:EGFP)* zebrafish. (**a–b**) Brightfield and fluorescence images of cultured cells (60 h after seeding) on a fibronectin coated culture dish without a preplating step (**a**) or after a 4 h preplating step (**b**). Black arrowheads, non-endothelial cells; BF- Brightfield. (**c**) Example of brightfield and fluorescence images (green for EGFP positive endothelial cells, and blue for nuclei (Hoechst 33342)) of isolated live ventricular cells on the preplate, after 4 h preplating. (**d**) Example of cultured cardiac endothelial cells after 1^st^ passage, stained for EGFP (green), rhodamine phalloidin (all cell types, red), and DAPI (nuclei, blue). (**e**) Quantification of endothelial cells to total cells after 1^st^ passage, showing high purity of the cultures. (n = 3, mean ± SEM). (**f**) RT-qPCR analysis using cardiomyocyte (*myl7*), fibroblast (*vim*), smooth muscle cell (*acta2*), epicardial (*wt1b*), and endothelial (*kdr*) markers (n = 3, mean ± SEM). Marker gene mRNA expression levels relative to *α-tubulin* were calculated using the ΔCt method (ct values are indicated in the Supplemental Table 2). Expression is relative to the individual marker’s expression in whole cardiac ventricles. All non-endothelial markers, besides *wt1b*, are depleted in the isolated cell population. Concomitantly, the isolated cells show enrichment for the endothelial marker *kdr*. One way ANOVA followed by Bonferroni’s post-hoc test (GraphPad Prism) was performed to evaluate statistical significance of differences. P < 0.05 was considered statistically significant. *** corresponds to P < 0.001.

The isolation and culture of cECs from adult organisms can provide a platform for screening small molecules^18^. Moreover, cells in culture are easy to image allowing the investigation of cell morphology and behavior for interesting mutant lines. The abundance (~37% of total cell population) of cECs in zebrafish makes the fish heart an ideal source of ECs for such a purpose. We performed 4–5 steps of 15 minutes collagenase digestion to isolate cells from 7–8 mpf *Tg(kdrl:EGFP)* ^*s843*^ zebrafish hearts (for detailed protocol see materials and methods and Supplementary Fig. S3), and isolated cells were seeded on fibronectin coated dishes (see Supplementary Fig. S4). We exploited the different attachment time required for the various cell types found in the heart to enrich the final cell population for cECs (Fig. 4a,b). Many non-ECs attach very quickly (3–4 h after seeding) (Fig. 4c); while CMs require a considerably longer time (24–48 h) than ECs (18–20 h). Thus, non-ECs are reduced in the final population with a 4 h pre-plating step (Fig. 4b). After pre-plating, the cell suspension, consisting mainly of cECs and CMs, was transferred to a fibronectin coated culture dish and incubated 18–20 h, to allow cEC attachment. Replenishing the culture medium at this time-point removes CMs that are still predominantly in suspension, resulting in a cEC enriched culture. To assess the purity of the isolated population, we fixed cultured cells after the 1^st^ passage and stained them using an anti-GFP antibody, rhodamine phalloidin, and DAPI. Our method consistently resulted in around 90% of the cells being cEC (*kdrl*:EGFP positive) in the final culture (Fig. 4d,e). Accordingly, non-EC specific marker gene expression, as evaluated by quantitative PCR, was strongly reduced while expression of the endothelial gene *kdr* was enriched after isolation (Fig. 4f). Thus, these primary cEC cultures from adult zebrafish ventricles provide a good system for *in vitro* studies.

### Characterization of cell cycle activity

**Figure 5:**
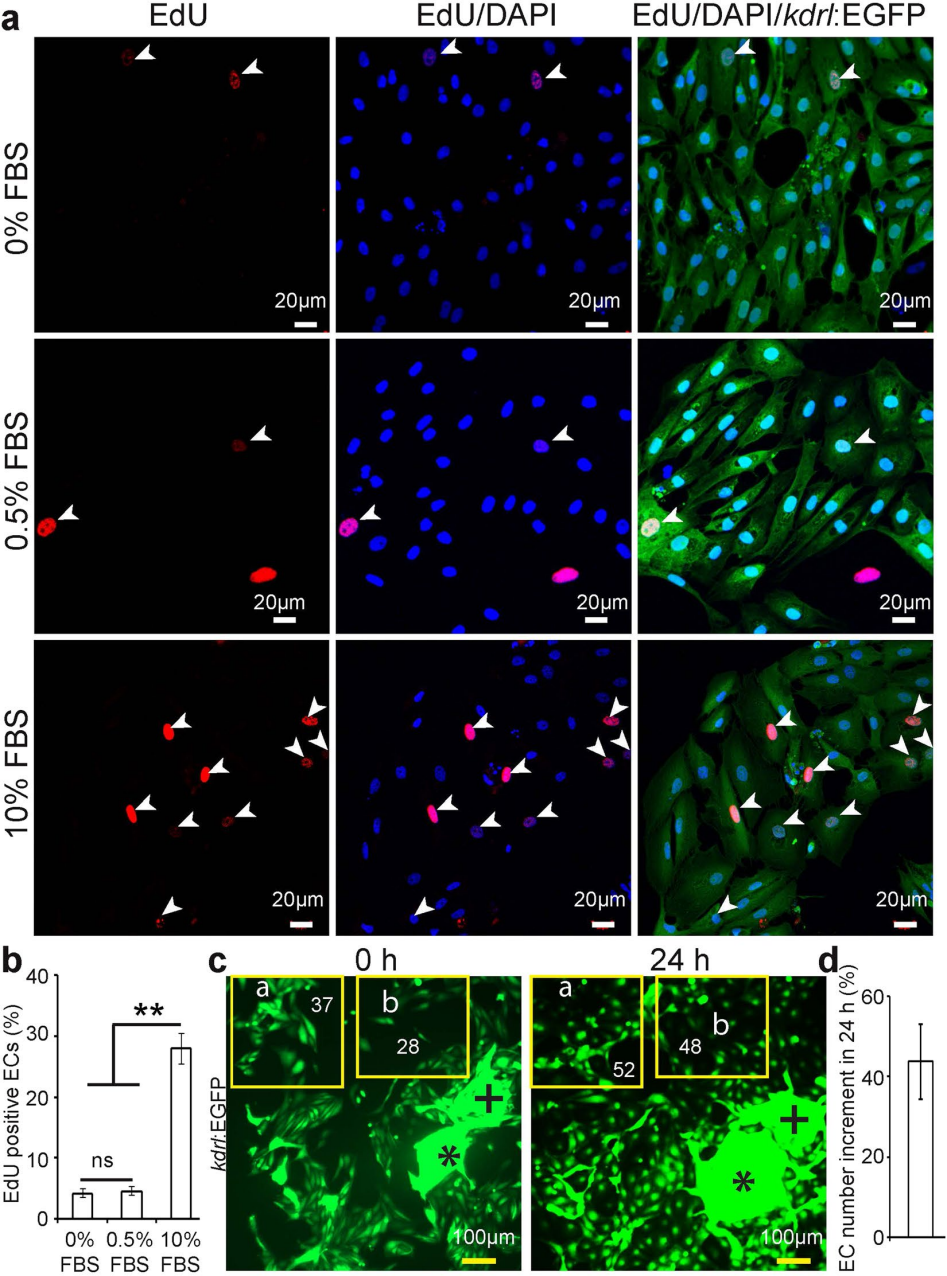
Cardiac endothelial cells proliferate *in vitro*. 24 h after seeding, ECs were cultured in EGM2 medium supplemented with 0, 0.5 or 10% FBS for 12 h and then analyzed for DNA synthesis using a 12 h pulse of EdU. (**a**) Representative images of endothelial cells stained for EGFP (green), EdU (red), and DAPI (nuclei, blue). White arrowheads point to EdU-positive endothelial cells. (**b**) Quantification of EdU+ EGFP+ endothelial cells without or with FBS in the culture medium (n = 3, mean ± SEM). P < 0.05 was considered statistically significant. (**c**) Example of cell density experiments. Cells were cultured with 10% FBS, resulting in endothelial cell proliferation. Plus and asterisk symbols are depicting landmarks, showing that pictures were taken from the same region for the cell density analysis. Yellow frames indicate areas of analysis. (**d**) Quantitative analysis of endothelial cell number increment in 24 h (n = 3, mean ± SEM). One way ANOVA followed by Bonferroni’s post-hoc test (GraphPad Prism) was performed to evaluate statistical significance of differences. P < 0.05 was considered statistically significant. ** corresponds to P < 0.05.

In order to use the cECs for *in vitro* experiments it is important to determine their cell cycle dynamics in culture. Isolated cECs were cultured on fibronectin coated glass coverslips using EGM2 medium with all supplements and 10% FBS. 33.90 ± 2.04% of cells had incorporated EdU after a 12 h pulse (see Supplementary Fig. S1f,g). To assess whether cell cycle activity was present in cECs in long term cultures, cells from a 2^nd^ passage were cultured on fibronectin coated glass coverslips using EGM2 medium with all supplements, either without or with FBS at two concentrations (0.5 or 10%). In the serum starved and 0.5% FBS treated ECs, 4.18 ± 1.18% and 4.56 ± 1.30% of the cells exhibited EdU incorporation after a 12 h pulse (Fig. 5a,b), indicating that 0.5% FBS fails to augment the basal cell cycle activity of cECs. However, stimulation with 10% FBS resulted in high DNA synthesis induction (27.96 ± 4.33%) (Fig. 5a,b). Taken together, the basal cell cycle activity of cECs in culture could be induced ~7 fold by adding 10% FBS to the medium.

To determine if DNA synthesis was followed by cytokinesis, we counted cECs in 10% FBS at days 1 and 2 (i. e. after 24 and 48 h of seeding respectively). Cell numbers rose by approximately 50% (Fig. 5c,d) indicating significant proliferation during this period.

### Scratch wound healing assay

**Figure 6:**
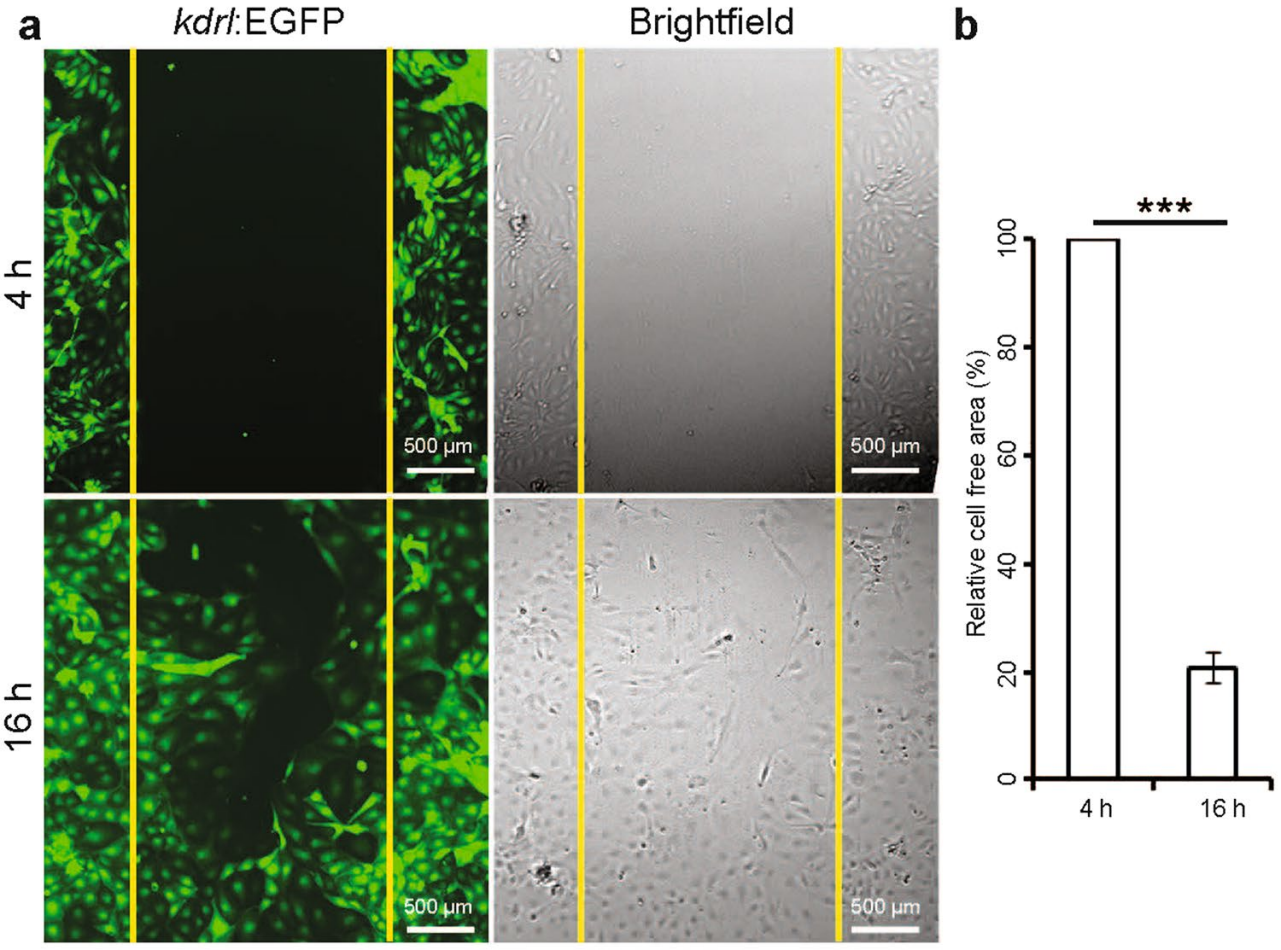
Scratch/wound-healing assay on cardiac endothelial cells. Assay was performed on isolated cardiac endothelial cells from adult *Tg(kdrl:EGFP)* zebrafish ventricles. (**a**) Fluorescence and brightfield images at 4 and 16 h after scratching. (**b**) Quantification of the initial cell free area covered by endothelial cell migration in 12 h. Cell free area at 4 h after scratch was considered as 100% (n = 3, mean ± SEM). One way ANOVA followed by Bonferroni’s post-hoc test (GraphPad Prism) was performed to evaluate statistical significance of differences. P < 0.05 was considered statistically significant. *** corresponds to P < 0.001.

The scratch induced wound healing assay is a well-established procedure to study cell migration *in vitro* ^19^. cECs were plated after a 2^nd^ passage in 48 well plates and left to establish a monolayer. After a 6 h serum starvation (in complete EGM2 medium without serum) step, the monolayer was disrupted using a P20 micropipette tip and the filling of the created gap was monitored using immunofluorescence and brightfield microscopy 4 and 16 h later (Fig. 6a). During these 12 h, 79.40 ± 5.78% of the initial cell free area became covered by cECs (Fig. 6b).

**Figure 7:**
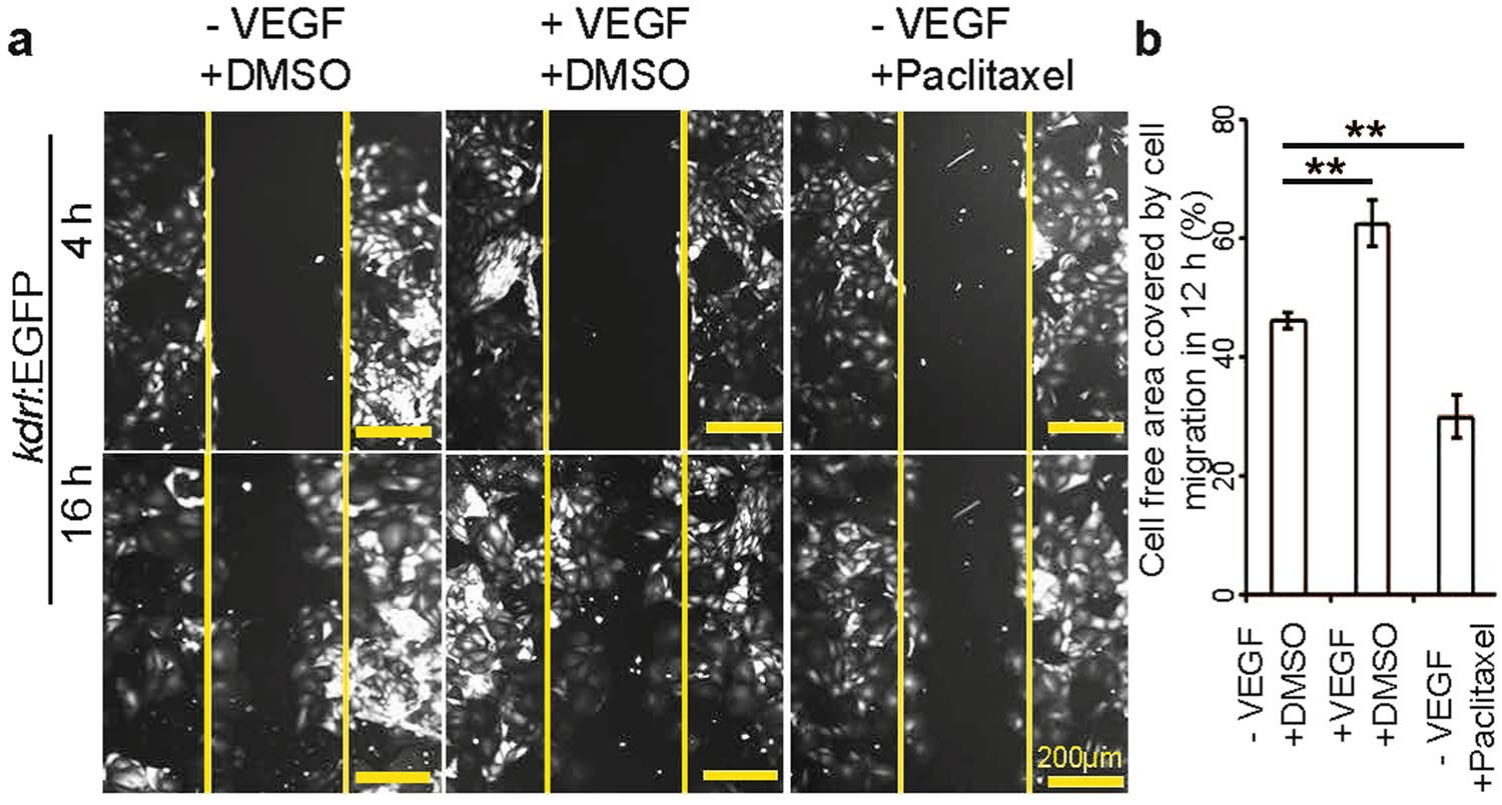
The migratory behavior of cardiac endothelial cells can be regulated by small molecule and growth factor treatment. (**a**) Fluorescence images at 4 and 16 h after scratching in the presence or absence of VEGF or Paclitaxel as indicated. (**b**) Quantitative analysis of the relative cell free area covered by endothelial cell migration in 12 h. Control cells were treated with DMSO, and in all cases, cell free area at 4 h after scratch was considered as 100% (n = 3, mean ± SEM). One way ANOVA followed by Bonferroni’s post-hoc test (GraphPad Prism) was performed to evaluate statistical significance of differences. P < 0.05 was considered statistically significant. ** corresponds to P < 0.05.

Next we sought to examine whether this assay could be a useful tool to screen small molecules that influence cEC migration. As proof of principle, we chose to test Paclitaxel, a microtubule stabilizer that inhibits endothelial cell migration^20^, and vascular endothelial growth factor (VEGF) that induces EC migration by promoting filamentous actin structure formation^21^. Cells from a 2^nd^ passage were cultured with 10% FBS to establish a monolayer and then serum and VEGF starved for 6 h. Subsequently, cells were treated with DMSO (control), or with a medium containing VEGF (same concentration as in EGM2), or with paclitaxel (12 nM final concentration). The cell monolayer was scratched 12 h post treatment and the cell free area was measured 4 and 16 h after the scratch. VEGF treatment significantly promoted cell migration in comparison to control; 62.34 ± 6.69% (45.98 ± 2.60% in control) of the cell free area was covered by cECs in 12 h, while, as expected, Paclitaxel treated cells covered only 29.85 ± 6.00% of the cell free area (Fig. 7). These data reveal that cECs are responsive to small molecule treatment, illustrating the value of this system for *in vitro* small molecule screening.

### Endothelial cell heterogeneity

The morphological and cell surface protein diversity of vascular EC populations among different vertebrate organs has been previously documented^2, 22, 23^. Here, we examined morphological features of zebrafish ECs from different tissues; the three cardiac chambers; atrium, ventricle, bulbus arteriosus (BA), and another highly vascularized organ, the gills. The average cell surface area of ventricular and atrial cardiac ECs was found to be similar and both populations included circular (circularity > 0.6) as well as elongated (circularity < 0.4) cells (see Supplementary Fig. S3). Although the average circularity of BA-ECs was similar to that of ventricular and atrial ECs, BA-ECs were generally ~4 times smaller than atrial and ventricular ECs (see Supplementary Fig. S3). Interestingly, ECs from gills were highly elongated (circularity < 0.2) and their average cell surface area was half that of atrial and ventricular ECs (see Supplementary Fig. S3). In conclusion, the intra- and inter-heterogeneity of ECs from different organs that can be sampled by cell isolation experiments may represent the adaptation and commitment of ECs to different functions in diverse organs.

## Discussion

Analysing and comparing the cellular composition of the adult zebrafish heart to the adult mouse heart which lacks regenerative potential is important to formulate different hypotheses and develop new strategies that could promote heart regeneration in mammals. Several studies have revealed the cellular composition of healthy adult mammalian hearts^6–8^. However, to date no such study has focused on the cellular composition of the adult zebrafish heart.

We (i) analyzed coronary vessel density in whole mount cardiac ventricles from 3 and 8 mpf zebrafish, (ii) estimated the EC and CM population in the adult zebrafish cardiac ventricle, (iii) compared cEC density in adult zebrafish and mouse hearts, and (iv) established a protocol to isolate and culture zebrafish cECs for *in vitro* studies.

We observed that zebrafish coronary vessels continue to grow after 6 mpf, which is considered the adult stage. This observation is in line with an earlier study where authors suggested that coronary vessel density in adult zebrafish continues to increase^17^. In the future it will be interesting to determine whether this continued growth is also observed in mammals. This finding paves the way for future work examining the correlation between the ability of coronary vessels to grow under physiological conditions and cardiac regeneration.

Based on electron microscopy analyses, Hu *et al*., suggested that at 3 mpf the zebrafish coronary vessels are subepicardial and trabeculae lack a coronary supply^24^. However, we found at 8 mpf in our immunohistological assays lumenized coronary vessel present throughout the compact layer. In addition, a few lumenized vessel-like structures were also visible in the trabecular layer. More specialized tools, including live angiography, will be needed to define whether these vascular structures in the trabecular layer are lumenized vessels or capillaries.

We also describe here, for the first time, the cellular composition of the adult zebrafish cardiac ventricle. It is well established that zebrafish hearts can regenerate^25^, but the mammalian heart shows fibrosis and remodeling after injury, possibly a result, at least in a part, of the high abundance of fibroblasts in mammalian hearts^6, 7, 26^. However, a recent report suggests that mouse cardiac ventricles comprise more than 43% ECs and a very small population of fibroblasts^8^, similar to what we observed in zebrafish. This discovery challenges the idea that the high fibroblast abundance could be inhibiting heart regeneration in mouse. Although, based on FACS analysis, the percentage of ECs in zebrafish and mouse hearts was similar; we found differences in the surface area coverage by ECs when looking at histological sections of zebrafish and mouse hearts. Technical details can affect the results of such histological measurements, as seen from inconsistent previous EC coverage estimates in mouse hearts^6–8^. Furthermore, in interspecies comparisons, other parameters such as degree of trabeculation, cell size and shape, or vascular organization can further complicate direct histological comparisons. Recently, Zhao et *al*. reported that in zebrafish Notch activation in endothelial and epicardial cells augments heart regeneration by inducing CM proliferation^5^. Moreover, another recent study proposed that fast revascularization is important for efficient heart regeneration in zebrafish^27^. Thus, it will be interesting to determine whether the larger relative surface area covered by ECs in zebrafish contributes to its efficient cardiac regeneration response. It is well established that ECs from different organs are diverse based on their morphology as well as the presence of membrane bound receptors^1, 2^. Thus, understanding the characteristics of tissue specific ECs will help develop strategies to treat organ specific pathologies. Here, using fluorescent transgenic reporter lines, we established a protocol to isolate cECs with high purity. To our knowledge, this is the first report of cardiac EC isolation without using EC specific antibodies, thereby circumventing their influence on the function of EC specific receptors *in vitro*. Morphological assessment of ECs isolated from atrium, ventricle, BA and gills supports the notion of EC heterogeneity in other vertebrate organs. We show that cECs are proliferative and respond to small molecules *in vitro*, highlighting their value in a small molecule screening platform for molecules with cEC migration or proliferation activity. This system will also be useful to perform *in vitro* loss- and gain-of-function experiments for candidate genes modulating cEC behavior.

## Electronic supplementary material

Supplementary Info
