# Supplementary material for: The zebrafish ventricle: A hub of cardiac endothelial cells for in vitro cell behavior studies
Source: Sci Rep. 2017 Jun 2;7:2687. doi: 10.1038/s41598-017-02461-1 (PMC5457396; doi:10.1038/s41598-017-02461-1)
Supplement: Supplementary file 1 — Supplementary Info [file 41598_2017_2461_MOESM1_ESM.doc]

**Title**

**The zebrafish ventricle: A hub of cardiac endothelial cells for *in vitro* cell behavior studies**

Chinmoy Patra^1,2a^*, Zacharias Kontarakis^2a^, Harmandeep Kaur^3^, Amey Rayrikar^1^, Debanjan Mukherjee^1^ and Didier Y. R. Stainier^2^

**Supplemental Table 1**

| Gene name | Gene symbol | primer sequence (5’-3’) | reference |
| --- | --- | --- | --- |
| *tubulin, alpha 1* | *tuba1* | F) CCTGCTGGGAACTGTATTGT  R) TCAATGAGTTCCTTGCCAAT | 1 |
| *kinase insert domain receptor* | *kdr* | F) CAAGTAACTCGTTTTCTCAACCTAAGC  R) GGTCTGCTACACAACGCATTATAAC | 2 |
| *myosin, light chain 7* | *myl7* | F) GGCTCTTCCAATGTCTTCTCC R) GGACTCCAGCTCTTCATCAC | 3 |
| *actin, alpha 2, smooth muscle, aorta* | *acta 2* | F) AAAGCAAGAGGGGAATCCTG  R) TCTCCCTGTTGGCTTTAGGA |  |
| *wilms tumor 1b* | *wt1b* | F) CCACACAGAAATGCCAAATG R) GACCCAGCACATCTTGTC | 3 |
| *vimentin* | *vim* | F) GGAAAAGAGCAAAGTGGAGGT  R) GATCTGCATCTCAGCAAGTTC | 3 |

**REFERENCES**

1.McCurley AT, Callard GV. Characterization of housekeeping genes in zebrafish: male-female differences and effects of tissue type, developmental stage and chemical treatment. *BMC molecular biology* 2008;**9**:102.

2. Hu G, Mahady GB, Li S, Hoi MP, Wang YH, Lee SM. Polysaccharides from astragali radix restore chemical-induced blood vessel loss in zebrafish. *Vascular cell* 2012;**4**:2.

3. Sander V, Sune G, Jopling C, Morera C, Izpisua Belmonte JC. Isolation and in vitro culture of primary cardiomyocytes from adult zebrafish hearts. *Nature protocols* 2013;**8**:800-9.

**Supplemental Table 2 : ct values for gene expression analysis Figure-4f**

|  | *tuba1* | *kdr* | *myl7* | *vim* | *wt1b* |  |  | *tuba1* | *acta 2* |
| --- | --- | --- | --- | --- | --- | --- | --- | --- | --- |
| Heart | 20.31 | 25.91 | 15.72 | 23.66 | 28.03 |  | Heart | 21.71 | 20.17 |
| EC1 | 21.75 | 25.02 | 26.00 | 28.05 | 29.14 |  | EC1 | 20.13 | 21.66 |
| EC2 | 20.50 | 24.44 | 21.57 | 26.82 | 27.98 |  | EC2 | 21.05 | 22.49 |
| EC3 | 20.77 | 24.68 | 24.43 | 26.70 | 28.66 |  | EC3 | 21.82 | 23.46 |
|  |  |  |  |  |  |  | EC4 | 21.59 | 21.7 |
| Heart | 23.25 | 27.71 | 17.24 | 25.31 | 30.24 |  |  |  |  |
| EC4 | 22.43 | 24.69 | 23.41 | 26.14 | 29.40 |  |  |  |  |

**
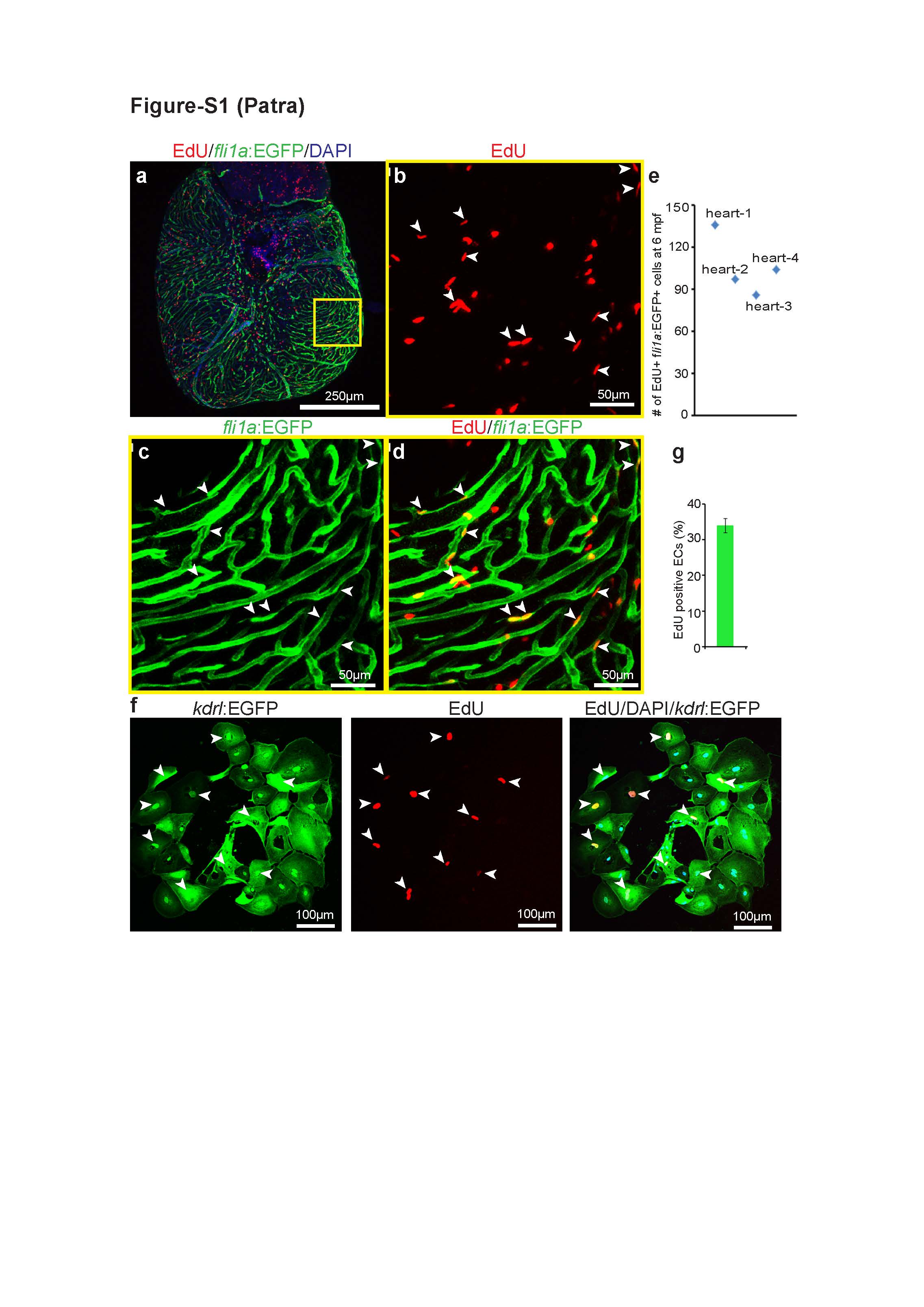
**

**
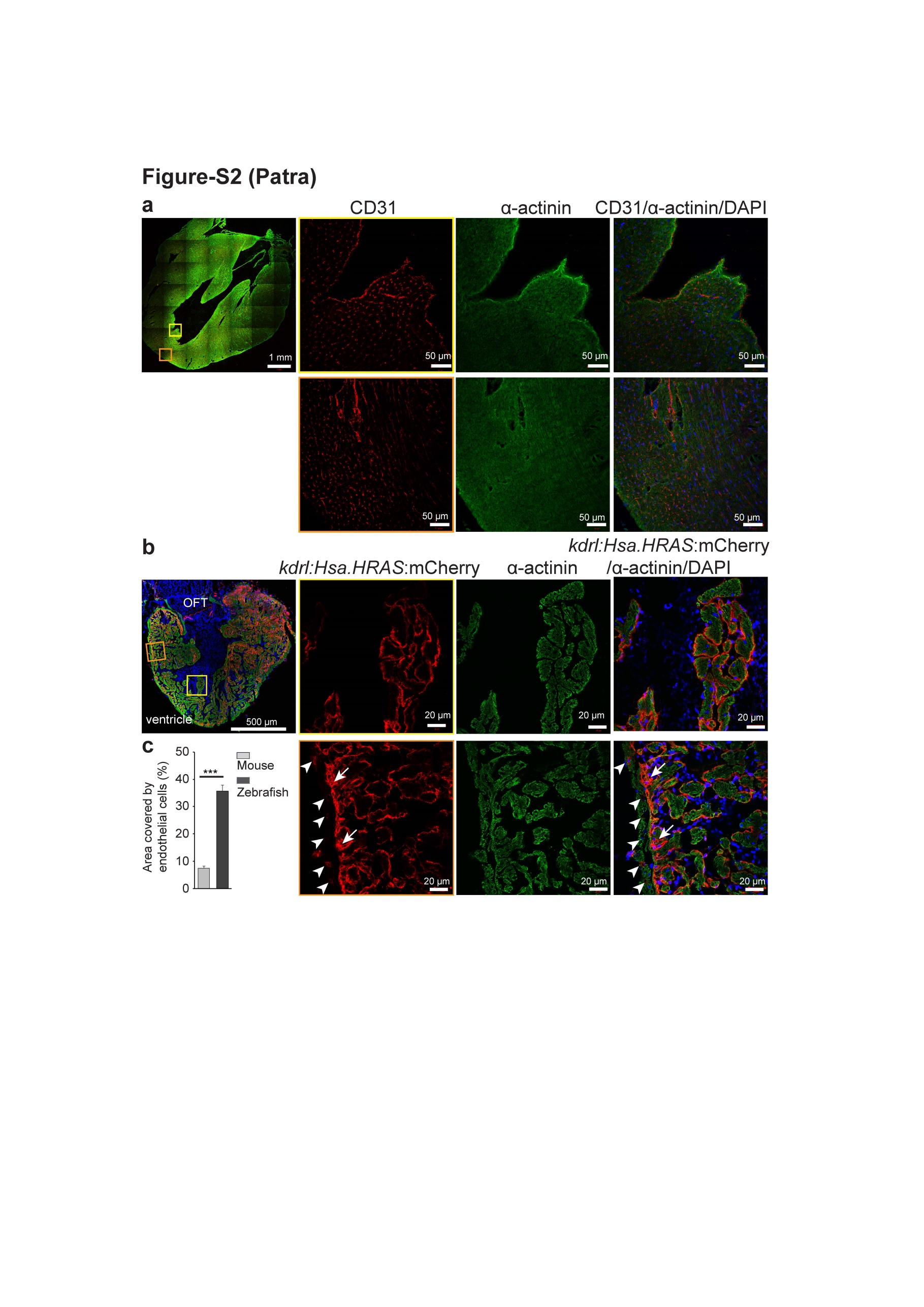
**

**
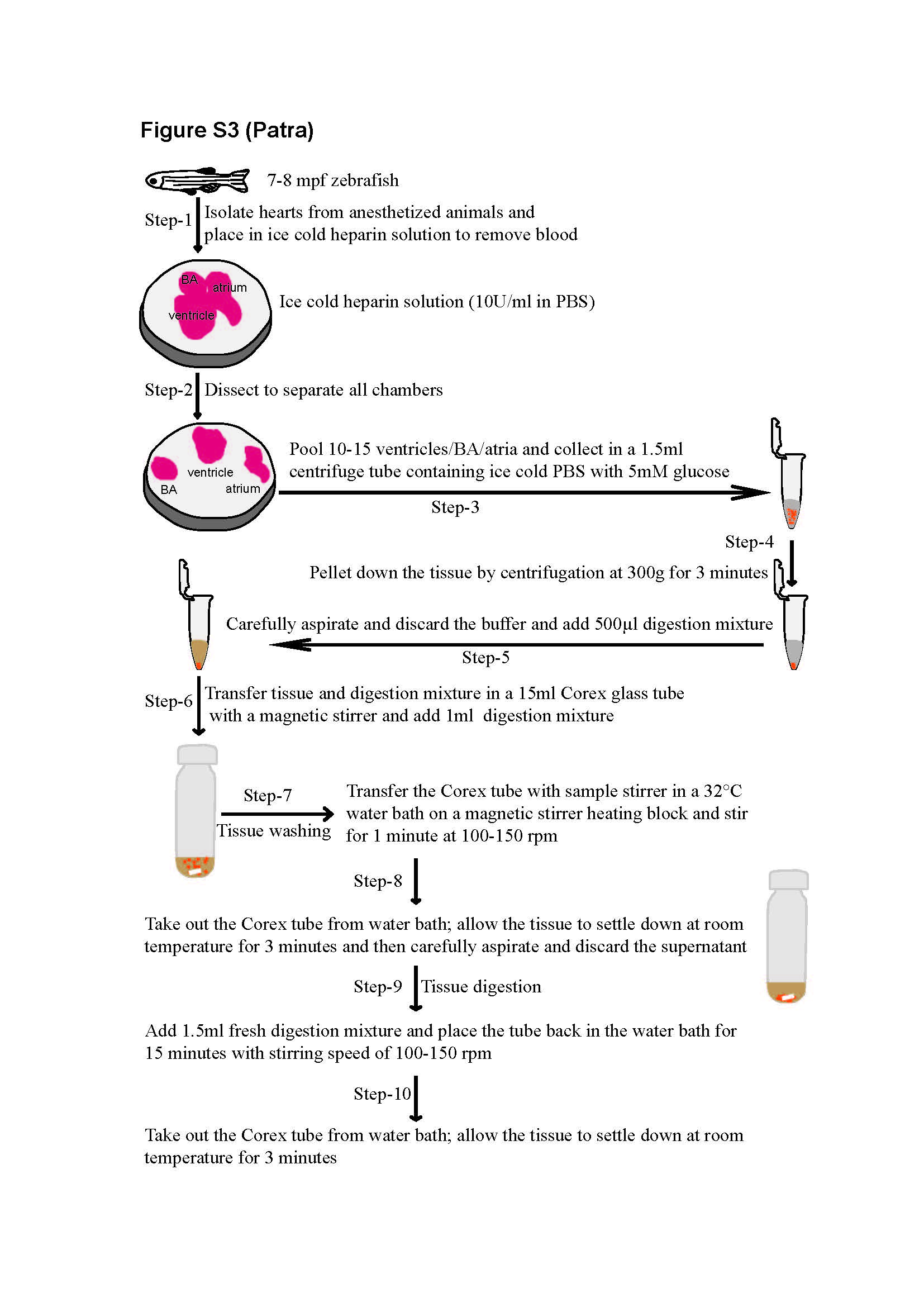
**

**
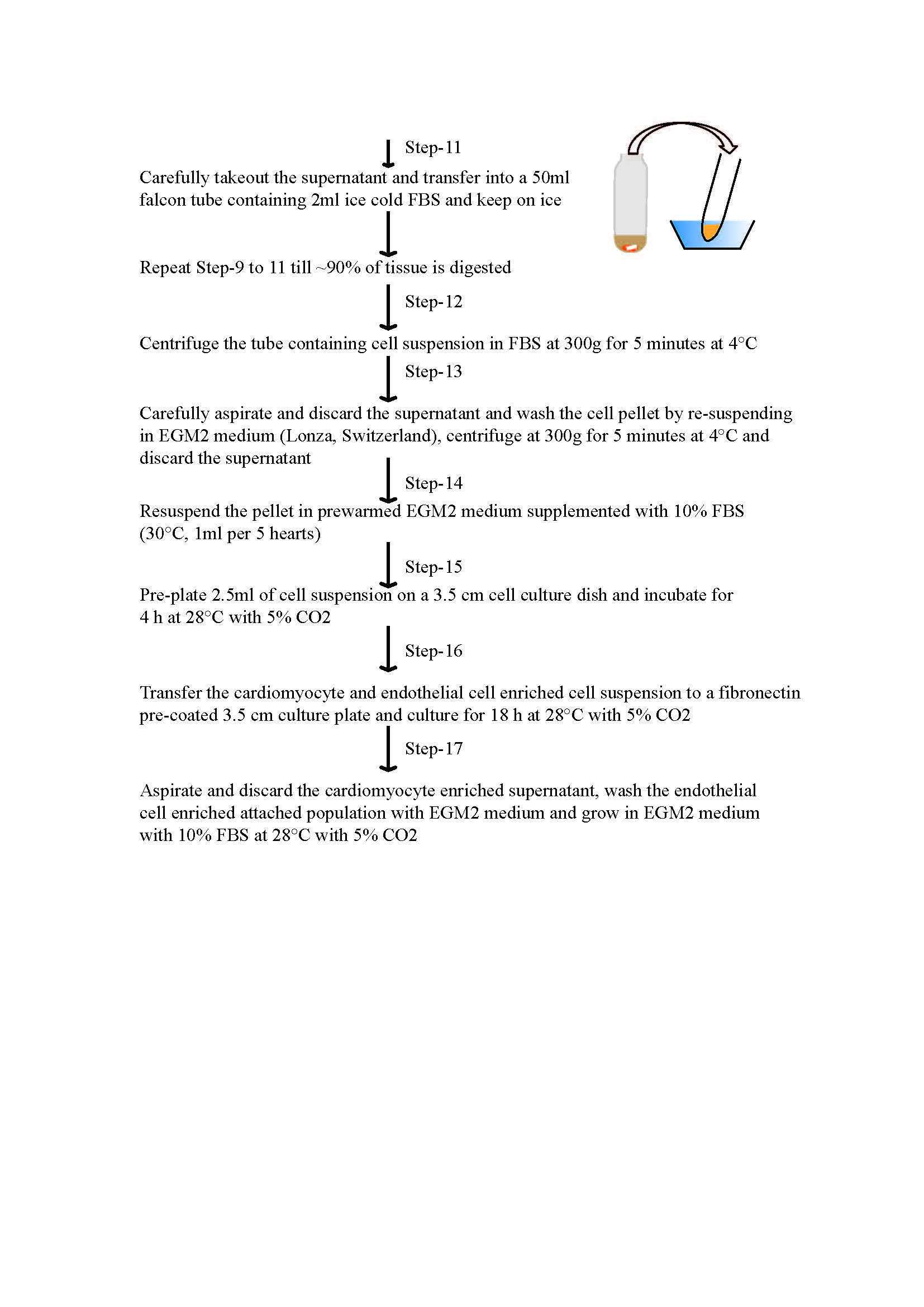
**

**
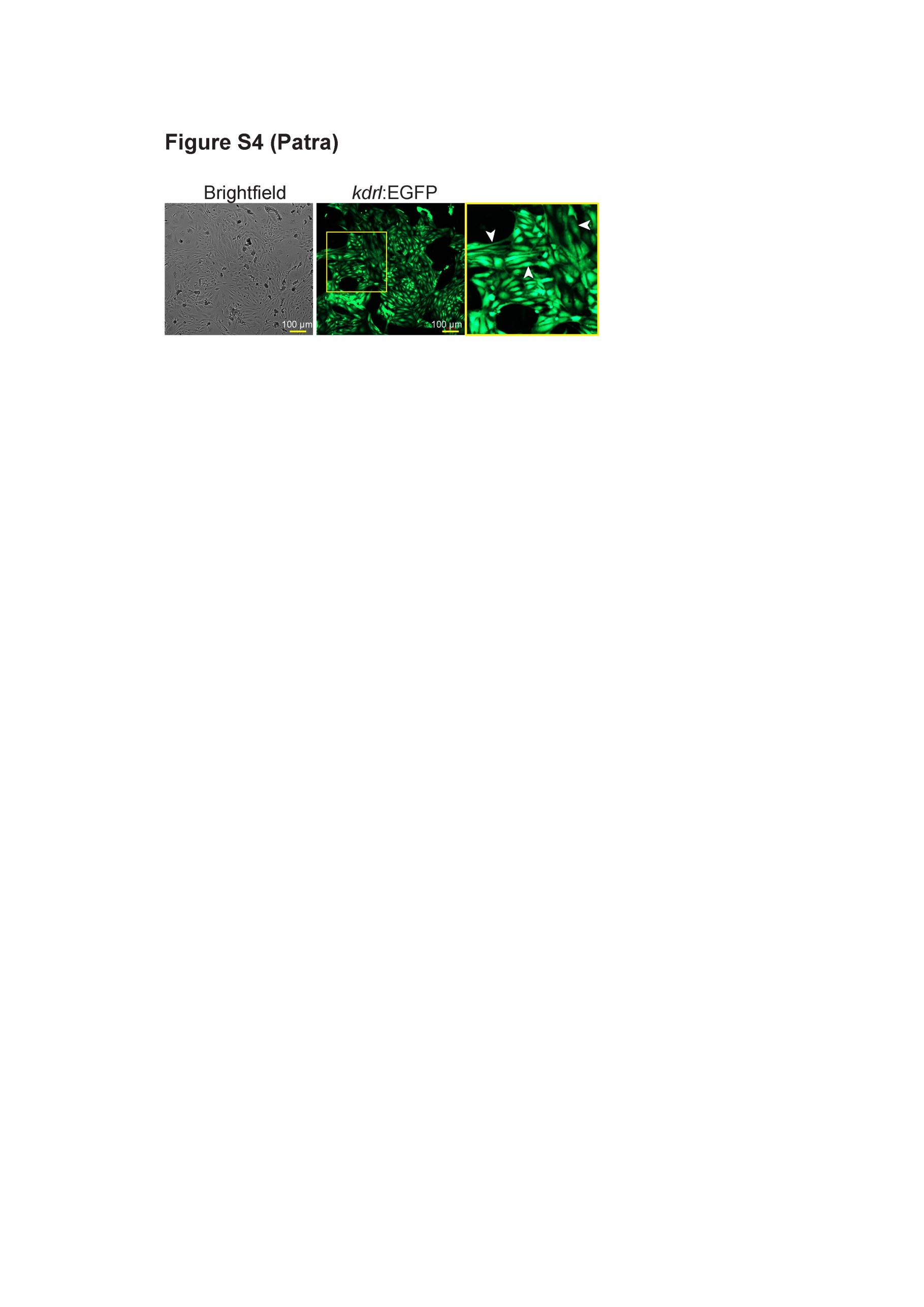
**

**
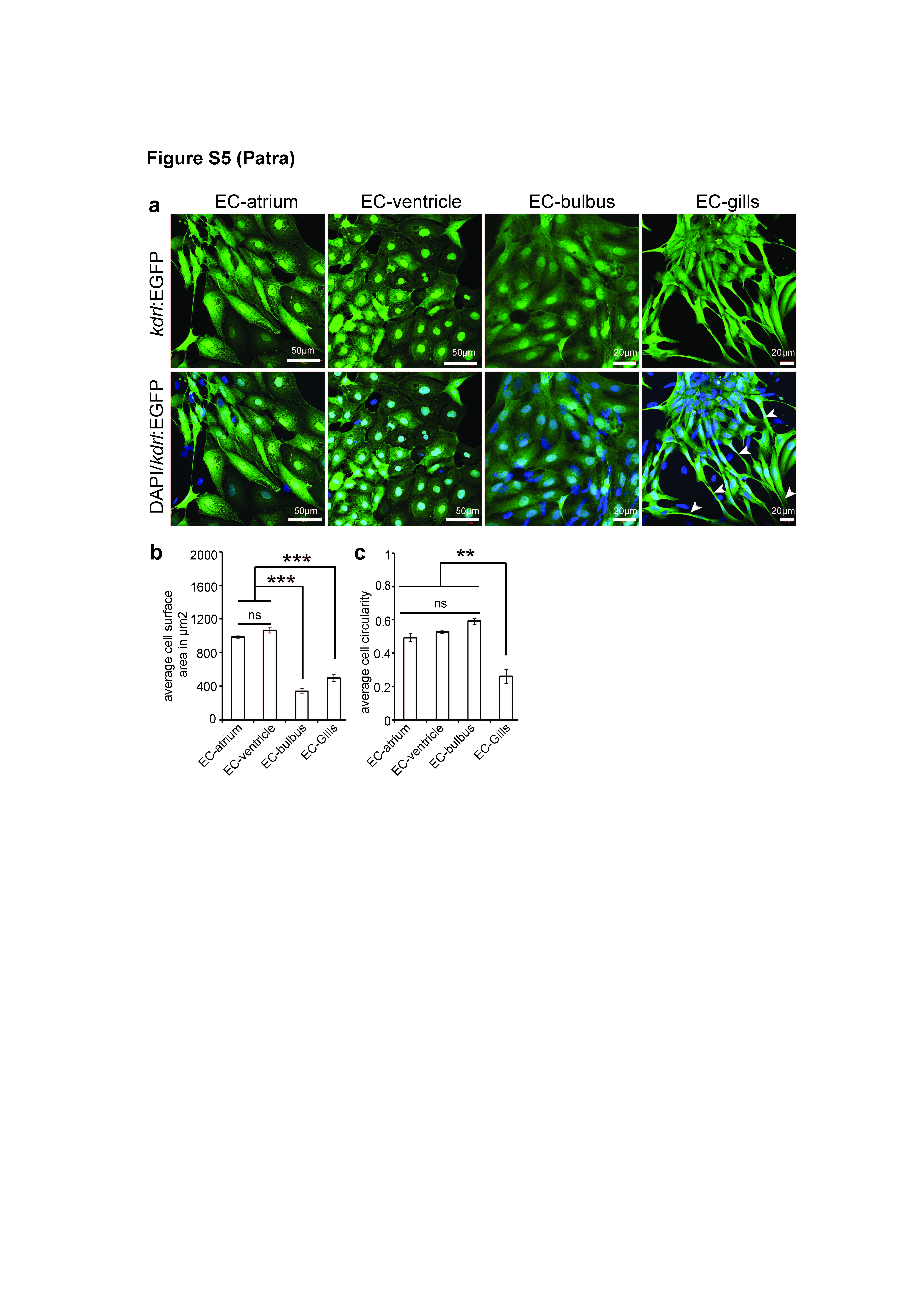
**

**FIGURE LEGEND**

**Figure S1: Cardiac endothelial cells are proliferative *in vivo*.** (a) Representative views of maximum confocal projections through 6 months old *Tg(fli1a*:EGFP*)* zebrafish whole mount hearts stained for EGFP (green), EdU (red), and DAPI (nuclei, blue). (b-d) Representative confocal images of ‘a’. Arrowheads point to EdU+ EGFP+ cells. (e) Quantification of EdU/EGFP-positive coronary endothelial cells. (n=4). (f) Representative views of cultured endothelial cells stained for EGFP (green), EdU (red), and DAPI (nuclei, blue). White arrowheads point to EdU-positive endothelial cells. (g) Quantification of EdU+ EGFP+ endothelial cells with 10% FBS in culture medium (n=3, mean±SEM).

**Figure S2: Endothelial cell density in 8 months old mouse and zebrafish ventricles.** (a) Representative confocal images of sagittal cryosections through 8 months old mouse hearts stained for α-actinin (green; marking cardiomyocytes), CD31 (red; marking the cell membrane of endothelial cells) and DAPI (blue; staining the nuclei). (b) Representative confocal images of sagittal cryosections through 8 mpf *Tg(kdrl:Hsa.HRAS*-mCherry*)* zebrafish hearts stained for α-actinin (green; marking cardiomyocytes), mCherry (red; marking endothelial cell membrane) and DAPI (blue; staining the nuclei). White arrowheads point to bigger coronary vessels in the outer compact layer; white arrows point to dense capillaries in the inner compact layer. (c) Quantification of the area covered by endothelial cells (as percentage of total cardiac tissue area). 10 sections from each heart from three zebrafish and three mice were analyzed (mean±SEM). One way ANOVA followed by Bonferroni’s post-hoc test (GraphPad Prism) was performed to evaluate statistical significance of differences. p < 0.05 was considered statistically significant. *** corresponds to P<0.001.

**Figure S3: Schematic depiction of endothelial cell preparation from adult zebrafish hearts.**

**Figure S4: Cardiac ventricular endothelial cells in culture**. Brightfield (BF) and fluorescence images of cardiac ventricular ECs after 58 h in culture; cell isolate was seeded on fibronectin coated cell culture dishes. White arrowheads point to ECs.

**Figure S5: Morphological diversity of endothelial cells from different tissues.** Cultured endothelial cells isolated from ventricle, atrium, bulbus arteriosus and gills. (a) Cells were stained for EGFP (endothelial cells, green), and DAPI (nuclei, blue) 2 days post seeding. White arrowheads point to elongated endothelial cells. (b, c) Quantitative analysis of the average surface area and average circularity of individual endothelial cells of different origins (n=3, mean±SEM). One way ANOVA followed by Bonferroni’s post-hoc test (GraphPad Prism) was performed to evaluate statistical significance of differences. P< 0.05 was considered statistically significant. *** corresponds to P<0.001 and ** corresponds to P<0.05.
